# Small Regulatory RNAs in Prokaryotes: Key Features, Identification, Environmental Roles, and Applications

**DOI:** 10.3390/microorganisms14071561

**Published:** 2026-07-16

**Authors:** Muhammad Ammar Nawaz, Muhammad Zohaib Nawaz, Syed Zeeshan Haider, Huda Ahmed Alghamdi, Wei Yan

**Affiliations:** 1 College of Marine Science and Technology, China University of Geosciences, Wuhan 430074, China; ammarbiotech15954@gmail.com; 2 School of the Environment and Safety Engineering, Jiangsu University, Zhenjiang 212013, China; zohaib@ujs.edu.cn (M.Z.N.); zaidiuaf@gmail.com (S.Z.H.); 3 Department of Biology, College of Sciences, King Khalid University, Abha 61413, Saudi Arabia; hudaghamdi@kku.edu.sa

**Keywords:** small regulatory RNAs, microbial adaptation, metabolic engineering, gene regulation, non-coding RNA

## Abstract

Small non-coding RNAs (sRNAs) are ubiquitous post-transcriptional regulators that enable rapid bacterial adaptation to fluctuating environments. Previous reviews have largely focused on sRNA mechanisms in model organisms. This review integrates computational prediction, meta-omics-based discovery, and synthetic biology applications of small regulatory RNAs in marine and environmental prokaryotes, providing a multi-layered perspective from identification to functional and engineering applications. The current landscape of sRNA identification tools is critically evaluated, with emphasis on strategies to overcome challenges such as false-positive predictions. Recent advances in mapping the RNA interactome and emerging evidence of previously underappreciated roles of sRNAs in environmental adaptation are discussed. Additionally, metagenomic and metatranscriptomic studies revealing the diversity of environmental sRNAs in uncultured microbial communities are summarized, highlighting their ecological significance. Finally, a curated overview of synthetic sRNA applications in metabolic engineering, including target genes and enhanced product yields, is provided as a resource for strain engineering. Collectively, this review provides a holistic view of prokaryotic sRNA biology, distinguishing it from more narrowly focused studies. Overall, sRNAs are highlighted as key regulatory elements linking microbial environmental adaptation with emerging biotechnological applications through advances in meta-omics guided discovery and synthetic RNA engineering.

## 1. Small Non-Coding RNAs and Their Classes

**Figure 1:**
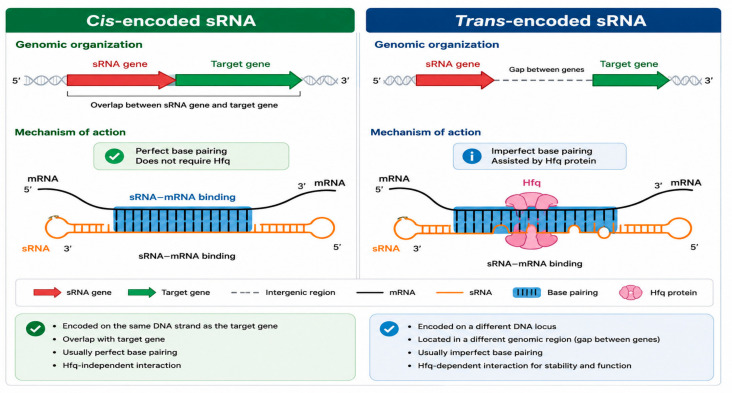
*Cis*- and *trans*-encoded sRNAs: genomic locations and mRNA interaction mechanisms.

Previously, non-coding RNA was misleadingly called junk RNA because it was considered non-functional [1]. Since the early 1990s, non-coding RNA has been increasingly acknowledged for its essential role in regulating gene expression [2]. In addition to model organisms, small regulatory RNAs play important roles in environmental and marine prokaryotes, where they contribute to adaptation under conditions such as salinity stress, hydrostatic pressure, temperature extremes, nutrient limitation, oxygen gradients, and heavy metal exposure. Among non-coding sequences, small regulatory RNAs (sRNAs), which comprise 50–500 nucleotides (nt), are recognized for their essential roles in important biological phenomena, including virulence, carbon metabolism, cold adaptation, and membrane permeability [3,4]. sRNAs, estimated to be present in the hundreds within bacterial genomes, function as key regulators or modulators of gene expression. They achieve this by binding to mRNA or protein targets, thereby influencing gene regulation at both the transcriptional and post-transcriptional levels. mRNA-binding sRNAs function as either *cis*- or *trans*-encoded sRNAs. *Cis*-encoded sRNAs are positioned adjacent to their target genes on the same strand, including riboswitches and RNA thermometers. RNA thermometers are *cis-*regulatory elements that modulate translation in response to temperature changes, whereas riboswitches regulate transcription or translation by binding small metabolites, providing distinct modes of gene regulation. Whereas RNA elements encoded on the opposite strands act as *trans*-encoded sRNAs (Figure 1). Although *cis*-sRNAs usually participate in the post-transcriptional inhibition of adjacent target mRNA, some that are involved in activating mechanisms have also been discovered. Earlier studies showed that *cis*-encoded sRNAs are primarily localized to plasmids, transposons, and phages. However, recent genome-wide transcriptomic and RNA-seq analyses reveal widespread chromosomally encoded *cis*-antisense RNAs across bacterial genomes, indicating that *cis*-regulatory RNAs are not limited to mobile genetic elements [5]. sRNAs were historically considered regulators of only fundamental processes, including transposition, mRNA degradation, suicide, replication, and translation initiation [6]. More recently, the role of sRNAs in environmental responses and adaptation has been increasingly recognized. For instance, five *cis*-encoded sRNAs have been reported to participate in cold adaptation in *Shewanella piezotolerans* WP3, highlighting their functional importance in environmental stress responses [7]. Most *trans*-encoded sRNAs require the host factor I protein (Hfq), an Sm-like RNA-binding chaperone, for target mRNA binding and sRNA stability [8] (Figure 1). In addition to Hfq, ProQ has emerged as another major RNA-binding chaperone involved in sRNA-mediated regulation, particularly in Gram-negative bacteria. *Trans*-regulatory sRNAs have been identified as pivotal players regulating diverse biological phenomena such as quorum sensing, iron homeostasis, and others in different bacteria [9]. Furthermore, some identified sRNAs regulate multiple targets by acting in both *cis* and *trans* manners, thereby blurring the distinction between these two classes [10].

## 2. Features, History, and Regulatory Roles of sRNAs

sRNAs in prokaryotes have certain characteristics that are useful for computational searches in various genomes. These features include a small size, the presence of multiple stem-loop structures, and typical localization in intergenic regions of genomes (between ORFs). In addition, sRNAs can originate from untranslated regions (UTRs) either on 5′- and 3′- ends. sRNAs derived from mRNA decay have also been discovered; an example is SroC, which base-pairs with GcvB and is derived from a locus encoding an amino acid ABC transporter. In addition to sRNAs derived from mRNA, some sRNAs originate from tRNA fragmentation [11]. These examples demonstrate that sRNAs exhibit greater functional complexity than previously recognized. Furthermore, many sRNAs in prokaryotes, particularly housekeeping sRNAs, are evolutionarily conserved among closely related species. For example, most expressed housekeeping sRNAs in *E. coli* are conserved in related organisms such as *Yersinia* and *Salmonella*. In contrast, adaptive sRNAs that respond to environmental stresses can evolve rapidly, exhibiting lineage-specific emergence and variation, reflecting their role in niche-specific adaptation [12].

The first sRNA was discovered by electrophoresis in *E. coli* in 1967 [13], and subsequent technological advances, including direct labeling, Northern blotting, microarray analysis, co-purification, and sequencing technologies have contributed to the discovery of a plethora of sRNAs in diverse prokaryotic genomes [13,14]. Most sRNA screening studies have focused on extensively investigated bacteria, such as *E. coli* [15], *Salmonella* [16], *Shewanella oneidensis* [17], *Shewanella piezotolerans* WP3 [7], *Listeria monocytogenes* [18], *Staphylococcus aureus* [19], *Pseudomonas aeruginosa* [20], and *Synechocystis* sp. PCC [21]. In this context, *E. coli* remained the most studied bacterium; approximately eighty sRNAs have been identified and validated in *E. coli* through experimental methods such as microarray and Northern blotting [22]. Of these 80 known sRNAs in *E. coli*, about 30 sRNAs are Hfq-dependent ones. Hfq is a member of the Sm-like protein family, which plays a key role in facilitating the interaction between sRNAs and their targets [23]. In *E. coli*, most sRNAs interact with the ribosome binding site located in the 5′-UTR of their target mRNAs [2], while others interact with coding sequences of the latter [24]. On the basis of computational prediction combined with experimental approaches, however, *E. coli* is thought more likely to code for 200 to 300 sRNAs [2].

Microarrays have been widely used to identify novel sRNAs. For example, RyhB, which is involved in iron homeostasis, was confirmed through microarray analysis. Another study used RNA sequencing to investigate how the sRNA MicF regulates OppA protein expression and maintains cell homeostasis [25]. Similarly, the elucidation of the sRNA genes expressed under specific conditions in *Bacillus subtilis* was predicted using comparative genomics-based methods and validation through DNA microarrays [26]. Some of these sRNAs also play important regulatory roles in response to perturbations in the external environment, therefore playing their role in adaptation.

sRNAs are not only present in bacterial genomes but have been acknowledged for their critical role as regulatory elements in archaea. The first sRNA discovered in archaea, CD box snoRNAs involved in RNA methylation, was initially found in *Pyrococcus* species and *Sulfolobus acidocaldarius*. The sRNA was first identified computationally using homology-based searches and then experimentally validated [27]. Advances in bioinformatics approaches for detecting sRNAs have facilitated the detection of sRNAs in the genomes of the hyperthermophile *Pyrococcus furiosus* and the methanogen *Methanocaldococcus jannaschii* [28,29]. Environmental and marine microbial communities represent major reservoirs of unexplored sRNA diversity. Meta-omics approaches have revealed that sRNAs in these environments are strongly associated with stress adaptation, particularly in uncultured microbial populations exposed to fluctuating salinity, pressure, and nutrient conditions. sRNAs can be packaged into extracellular vesicles, allowing communication between microbes and their hosts. These extracellular sRNAs regulate gene expression, metabolism, and stress responses across microbial communities, facilitating adaptive bacteria–host interactions.

New experimental methods, such as RNomics, co-purification of sRNAs, and high-throughput sequencing, have been developed to complement bioinformatics prediction approaches. These technological advancements have resulted in the identification of sRNAs in model archaeal species, including *Methanosarcina mazei* [30], *Sulfolobus solfataricus* [31], *Archaeoglobus fulgidus* [32], and *Haloferax volcanii* [33]. Based on such studies, the number of sRNAs in archaea is believed to be comparable to that of bacteria.

Next-generation sequencing and bioinformatics approaches have demonstrated that intergenic regions play pivotal regulatory roles in organismal transcription. sRNAs facilitate the adaptation of the microbes in challenging environments by mediating responses to perturbations in their external environment and modulating stress responses and metabolic pathways. The bacterial regulatory network is thus much more complex than previously realized. sRNA-mediated regulation offers distinct advantages over other mechanisms, including reduced metabolic burden and rapid gene expression control. The regulatory advantages of sRNAs over protein-based regulators are increasingly recognized. sRNAs enable bacteria to respond to environmental signals more rapidly and with fewer resources by bypassing the translation stage, while their incomplete complementarity to target RNAs allows for flexible, multi-target regulation in response to diverse cellular signals, making them particularly well-suited for mediating adaptation to stressful conditions [34]. sRNAs thus enable microbes to survive and thrive in harsh, unfavorable environments. The sRNA’s function in carbon and amino acid metabolism and metal sensing has been reported previously [35]. sRNAs also help the adaptation of microbes in challenging environments by participating in related phenomena like quorum sensing systems and biofilm development. Furthermore, sRNAs participate in mediating responses to different stresses and harsh conditions. Briefly, sRNAs are key players having diverse roles in important physiological processes and regulatory mechanisms. Recent studies have revealed that chemical modifications of sRNAs, including methylation and pseudouridylation, can modulate their stability, target recognition, and regulatory efficiency. This layer of post-transcriptional control, known as epitranscriptomic regulation, adds further complexity to sRNA-mediated gene regulation and highlights additional mechanisms by which bacteria and archaea adapt to environmental stresses. sRNAs exhibit remarkable evolutionary plasticity across prokaryotic taxa. While a subset of sRNAs is highly conserved among closely related bacterial species, many others show lineage-specific emergence, reflecting adaptation to distinct ecological niches. Horizontal gene transfer has contributed to the dissemination of certain regulatory RNA elements across bacterial and archaeal taxa, enabling rapid acquisition of regulatory functions in new environments. In addition, sRNAs undergo adaptive evolution driven by environmental pressures such as nutrient limitation, temperature shifts, and host-associated interactions. This combination of conservation, innovation, and horizontal transfer highlights the evolutionary flexibility of sRNAs and their central role in microbial adaptation.

In contrast to mRNA-binding sRNAs, some regulatory RNAs function by directly binding to proteins and modulating their activity. The well-known example of protein-binding sRNA is 6S RNA, which is approximately 180–200 nt in size and evolutionarily conserved across bacterial lineages. The secondary structure of 6S RNA enables it to bind specifically to the housekeeping RNA polymerase (RNAP) holoenzyme (σ^70^-RNAP in *E. coli*) [36]. This interaction sequesters RNAP, inhibiting transcription from a subset of σ^70^-dependent promoters and facilitating the transition from exponential growth to the stationary phase. Crucially, 6S RNA levels are dynamically regulated in response to bacterial growth phases. Its abundance significantly increases as bacteria transition from exponential growth to the stationary phase, reaching peak levels during nutrient limitation and stress conditions. This inverse relationship between 6S RNA levels and growth rate maximizes RNAP sequestration during stationary phase and stress, enabling resource conservation and environmental adaptation. Conversely, during rapid exponential growth, lower 6S RNA levels permit efficient transcription of proliferation-associated genes. This dynamic regulation establishes 6S RNA as a critical regulator of bacterial growth-phase transitions and stress responses [37]. The regulatory cycle is completed by the synthesis of short product RNAs directly on the 6S RNA template; when pRNAs reach a critical length (≥12–14 nt), they trigger the release of RNAP from the complex, restoring transcriptional activity [38].

Recent studies have revealed that 6S RNA-mediated regulation is critical for bacterial survival under specific stress conditions. For example, deletion of the 6S RNA gene in *Rhodobacter sphaeroides* resulted in impaired growth under high salt stress (250 mM NaCl), with elevated expression of the downstream *sspA* gene encoding a putative membrane protein involved in salt adaptation [39]. Similarly, 6S RNA deletion in *E. coli* led to a lethal phenotype upon exposure to high concentrations of hydrogen peroxide (10–20 mM), with reduced expression of key oxidative stress genes such as *ahpC*, *sodA*, and *tpx* [40]. *Bacillus subtilis* exhibits two copies of 6S RNA, and deletion of both the paralogs (6S-1 and 6S-2) led to accelerated cell density decline in the stationary phase and enhanced outgrowth under alkaline stress (pH 9.8) [41]. These findings establish 6S RNA as a crucial regulator of bacterial stress responses, mediating through a mechanism fundamentally distinct from the base-pairing sRNAs discussed in previous sections. 6S RNA represents a distinct class of regulatory RNA that interacts with the RNA polymerase σ70 holoenzyme, thereby modulating transcription during growth-phase transitions. Its levels increase during stationary phase and stress conditions, enabling global transcriptional reprogramming. Upon nutrient recovery, short product RNAs are synthesized from the 6S RNA template, which facilitates release of RNA polymerase and restoration of transcriptional activity. This mechanism differs from classical base-pairing sRNAs and highlights the diversity of RNA-mediated regulation in bacteria.

The functional diversity of sRNAs is highlighted by providing a few examples in different bacterial species (Table 1). For example, the sRNA StsR from *Rhodobacter sphaeroides* regulates the *dcw* gene cluster in an UpsM-dependent manner [42], while IhtA from *Chlamydia trachomatis* targets *hctA* and *ddbA* to influence elementary body development, playing their role in cell division [43]. Similarly, PcrX from *R. sphaeroides* modulates *pufLMX* expression in an Hfq-dependent manner [44], and IsaR1 from *Synechocystis* 6803 targets *upp* and *crtH* during iron starvation, thereby demonstrating an important role in photosynthetic bacteria [45].

Temperature adaptation is exemplified by five *cis*-sRNAs (cis4, cis5, cis6, cis10, and cis15) identified in the deep-sea bacterium *Shewanella piezotolerans* WP3, which target *ompA*, amidohydrolase, dioxygenase, and PAS domain-containing genes during cold adaptation [7]. Similarly, sRNAs RyjB and 6S RNA play their important role in acid stress responses by targeting *phoP* [59] and *pspF* [60] genes, respectively, in *E. coli*. In case of countering oxidative stress, RsaC s’ role by modulating *sodA* and the MntABC system via RNase III has been discussed previously in *Staphylococcus aureus* [61]. Similarly, under osmotic stress, role of RyfA in regulating the *rpoH* in a σ^32^-dependent manner [62] and RprA in modulating *rpoS* through Hfq and RNase III [63], has been studied earlier. Aerobic and anaerobic transitions are mediated by FnrS in *Sphingopyxis granuli*, which targets *fnr* [64]. Similarly, in *E. coli*, sRNAs including OmrA/OmrB regulate four porins with Hfq assistance [65], RybB targets *omp*mRNAs under σ^E^ control [66], and RprA again contributes to *rpoS* regulation [63]. Numerous sRNAs associated with virulence has been reported in earlier studies such as STnc150 from *Salmonella typhimurium* targets *fimA* [67], MTS1338 from *Mycobacterium tuberculosis* regulates *furA*, *whiB4*, and *phoP* [68], sRNA23 from *Streptococcus suis* affects FBA and *rplB* [69], RyfA1 from *Shigella dysenteriae* targets *ompC* with Hfq involvement [70], FasX from *Streptococcus pyogenes* regulates the *fasBCA* operon [71], RivX from *Clostridium perfringens* modulates *mga* through *rivR* [72], and RsmY-RsmZ in *P. aeruginosa* control T3SS and *pel* via the HptB/RetS pathway [73]. Finally, plasmid and phage defense systems employ sRNAs such as CopA from *S. aureus*, which targets *copT* with Hfq assistance [74]. Similarly, Sok from *E. coli* regulates *hok* [75], and crRNAs from *Campylobacter jejuni* target cellular RNAs in CRISPR-mediated defense [76].

sRNAs regulate diverse physiological processes and regulatory mechanisms spanning the entire bacterial cell cycle. A recent comprehensive study demonstrated the remarkable versatility of bacterial ncRNAs, documenting their involvement in fundamental processes including replication, transcription, translation, energy metabolism, antibiotic resistance, and virulence [77]. Table 1 summarizes the versatile functions performed by sRNAs in different bacterial species. As plenty of sRNAs have been discovered in diverse prokaryotic species related to almost every global response.

## 3. Discovery of Environmental sRNAs

Most sRNA identification studies, as discussed above, have been performed and remain focused on a few model microorganisms. Because almost all previous sRNA discoveries have focused on culturable microorganisms, environmental sRNAs from uncultured organisms, which comprise more than 99% of all microbes, remain largely unidentified. Consequently, information on sRNA diversity and ecological relevance in various environments, such as deep-sea microbial communities, is limited. The identification of sRNAs in uncultured microbes recovered from various environments should provide insights into their ecological and functional diversity. Metagenomics offers culture-independent approaches to explore the community structure and function of uncultured microbes. Metagenomics-based strategies have recently been used to reveal genomic reads and analyze the diversity of microbial species in various environments, such as the gut of animals, including humans, and deep-sea samples [78,79]. Nevertheless, metagenomics lacks the capability to provide insights into the physiology of microorganisms and their adaptation mechanisms. Metatranscriptomics complements metagenomics by revealing differential gene expression under varying environmental parameters.

Meta-omics studies have identified numerous sRNAs from diverse environmental samples. These studies have reported the presence of several sRNAs belonging to diverse classes of sRNA in deep-sea samples. These sRNAs play a role in regulating microbial activity in their natural habitat [79]. Additionally, 24 sRNAs, including highly expressed sRNA Yfr28, were discovered in low-nitrogen conditions in oceanic environmental populations through a metatranscriptomic approach [80]. Another study reported 2181 uncharacterized putative candidate sRNAs in deep-sea samples from a hot spring using a metatranscriptomic approach, revealing novel associations between the anticodons of tRNAs and tRNA degradation sites [81]. At the HOT Station ALOHA site, 18,000 to 47,000 sRNAs belonging to 66 different groups were identified at various depths using metatranscriptomics. Depth-dependent variations in psRNAs (putative sRNAs) were observed, along with their involvement in carbon metabolism and nutrient acquisition [82]. In the human oral samples, 12,097 sRNAs were identified using metatranscriptomics, which might be playing their role in maintaining the activity and balancing the oral microbiome [83]. Similarly, 19 sRNAs were detected in blood plasma using Illumina and sRNA isolation columns, revealing the presence of sRNAs transcribed by the microbes in blood [84]. In the human gut, 208 candidate sRNAs were identified using metagenomic and metatranscriptomic approaches, showing significant differences between in vitro and in vivo gene expression [85]. Another study identified 59 sRNAs in the human gut using a metatranscriptomic approach, revealing the physiology of the complex gut microbiota and detecting small RNAs crucial for prokaryotic physiology and pathogenicity [86]. Lastly, in the halite nodules of the Atacama Desert, 155 conserved sRNAs, 925 antisense sRNAs, and 613 intergenic sRNAs were identified through metatranscriptomics, elucidating the functions and mechanisms of sRNAs therein [87]. Key findings from various studies on environmental sRNAs have been provided in Table 2. Recent studies have highlighted the direct role of sRNAs in regulating the key biogeochemical processes. For example, Moeller and coworkers [88] provided the current knowledge on sRNAs participating in the nitrogen cycle, including nitrogen fixation (e.g., *nfsS* in *Pseudomonas stutzeri*), nitrogen assimilation (e.g., *nsiR4* in cyanobacteria, multiple sRNAs in the archaeon *Haloferax mediterranei*), and denitrification (e.g., *denR* in *Paracoccus denitrificans*). Despite these advances, a major challenge in metatranscriptomic identification of sRNAs is distinguishing true regulatory sRNAs from RNA degradation products. Fragmentation during RNA processing, environmental RNA decay, and library preparation biases can generate short RNA reads that mimic sRNA signals. Although current approaches integrate size selection, strand specificity, secondary structure prediction, and conservation patterns, multiple technical challenges limit environmental sRNA discovery: contamination from environmental nucleic acids during sampling and sequencing; annotation difficulties from incomplete reference genomes for uncultured organisms; reduced sensitivity for detecting low-abundance transcripts; and insufficient experimental validation linking identified sRNAs to physiological functions. These limitations collectively create a significant gap between computational prediction and functional characterization in environmental systems.

## 4. Computational Methods for Identifying sRNAs

Before the availability of bioinformatics methods for sRNA prediction, biochemical approaches largely overlooked sRNAs. This neglect was due to various reasons, including the fact that sRNAs are poor targets for mutational screenings because they do not encode proteins and are insensitive to frameshifts and nonsense mutations. Most of the sRNAs discovered so far are found in intergenic regions (IGRs) [89,90]. Compared with other genomic regions, IGRs are under low selection pressure and thus have more opportunity to evolve in fluctuating environmental conditions. Another reason for previous experimental inattention to sRNAs is that such methods are time-consuming and tedious. Moreover, sRNAs are expressed under very specific conditions [91], hindering their validation using experimental validation. Therefore, all the positively predicted sRNAs cannot be validated using experimental approaches. Currently, available sRNA prediction algorithms have given rise to efficient, large-/genomic-scale computational screening of sRNAs as a complement to experimental methods [92]. Hör and coworkers highlighted that the computational guided integrative approaches linked with protein co-immunoprecipitation studies (e.g., with Hfq) have been proven successful and expanded the known repertoire of sRNAs in well-studied bacteria, including *E. coli* and *Salmonella* [93]. Previous computational searches have estimated that, on average, a eukaryotic genome contains thousands of regulatory RNAs, whereas only a few hundred exist in prokaryotic genomes [94]. Although the predicted abundance of sRNAs across various genomes is significantly greater than previously expected, the functions of most are yet to be elucidated. Nevertheless, many sRNAs have been recently found to play a pivotal role in the adaptation of organisms to various environmental conditions [95]. These discoveries demonstrate that sRNAs are critical components of regulatory networks that mediate gene expression in response to changing environments. Summaries of current knowledge on sRNAs in different organisms are available in online databases (https://rfam.org/; www.noncode.org; https://biobases.ibch.poznan.pl/ncRNA, accessed on 20 May 2026).

**Figure 2:**
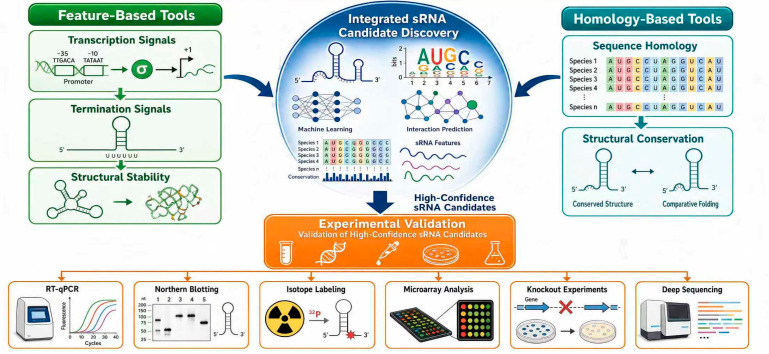
Combined computational and experimental approaches for identification of sRNAs in bacterial genomes.

The recent development of computational algorithms based on features including thermodynamic stability of their secondary structures, sequence and structural conservation, termination signals, lack of open reading frames, and non-coding sequence homology in related genomes [93,96] has provided an opportunity to efficiently perform genome-wide screening for sRNA prediction in various microbial genomes. A few of these comparatively reliable tools (Table 3) include QRNA [97], RNAalifold [98], RNAz [99], sRNAscanner [100], and NAPP [101]. For instance, comparative evaluations have demonstrated that RNAz-based pipelines combined with RNA secondary structure prediction tools provide a better balance between precision and recall compared to standalone approaches. These tools have inherent advantages and disadvantages. For example, some predict sRNAs based on a single feature, such as RNA structural stability and rho-independent termination sites, whereas others rely on sequence and structure conservation. Integrated computational pipelines combining sequence-based and structure-based tools have shown improved prediction accuracy. For example, combinations such as RNAz + RNAfold + RNAalifold or QRNA + RNAz have been widely used to improve sensitivity while reducing false-positive rates in bacterial sRNA prediction. Benchmarking studies have also reported that ensemble approaches integrating thermodynamic stability and comparative genomics outperform single-tool predictions in both precision and recall. Despite these advances, computational prediction tools still exhibit trade-offs between sensitivity and specificity, with highly sensitive models often increasing false-positive rates, while highly specific approaches may miss divergent sRNAs in environmental and non-model organisms. This limitation is further amplified in metagenomic datasets due to sequence fragmentation and uneven coverage, highlighting the need for continued refinement and standardized benchmarking of prediction algorithms. Finally, predicted sRNA could further be subjected to experimental validation using RT-PCR, Northern blotting, knockout experiments, and isotope labeling methods (Figure 2).

Although experimental validation techniques are theoretically available, three interconnected obstacles practically prevent confirmation of computationally predicted sRNAs in environmental systems. First, environmental sRNAs exist at or below detection thresholds, either due to genuinely low abundance or conditional expression triggered only by specific environmental stimuli. Second, environmental RNA samples are inherently unstable, degrading rapidly during collection and processing, thereby compromising both transcript recovery and analytical accuracy. Third, the inability to culture most environmental microorganisms eliminates classical genetic validation approaches, such as knockouts and reporter assays, forcing researchers to rely on correlative analyses without direct functional verification. Together, these barriers establish a substantial gap between successful computational prediction and actual experimental validation in natural microbial communities. Furthermore, incomplete or poorly characterized regulatory machinery in certain environmental species limits functional validation of predicted sRNAs. These limitations collectively result in a significant gap between computational prediction and experimental confirmation in environmental sRNA studies. Only a few studies have reported the large-scale sRNA prediction and characterization that have individually identified over 100 sRNAs (Table 3). In an investigation of *Pseudomonas aeruginosa*, 2759 sRNAs were predicted [102]; however, only 31 were subjected to experimental verification, and 17 were finally confirmed. Khoo et al. [92] tested 15 out of more than 1300 computationally predicted sRNAs in *Burkholderia pseudomallei*, of which only eight were finally confirmed. In most of the previous studies authors choose a fraction of sRNAs they are particularly interested in, out of all computationally predicted for further validation through experiments. Different computational tools have different sensitivities, with the number of generated putative sRNA ranging from hundreds to thousands. The integration of multiple tools, however, can yield a reasonable number and a more reliable dataset for prediction.

The proportion of confirmed sRNAs from the tested candidates in previous studies was as high as 100%, in *Synechocystis* sp. PCC6803 [104], *B*. *cenocepacia* J2315 [105], *P. mirabilis* [107], *S. pneumoniae* D3 [108] *Prochlorococcus* stains NATL2A and MED4 [80], *B. pertussis* [110], *S. mutans* [111], and *H. somni* [112], or as low as 29% in the case of *Staphylococcus aureus* [103] (Table 3).

### Computational Approaches and Bottlenecks in Environmental sRNA Analysis

Despite significant advances in computational prediction of small regulatory RNAs, several bottlenecks remain in the analysis of environmental sRNAs. One major limitation is the high rate of false-positive predictions due to short sequence length, low sequence conservation, and overlap with non-functional RNA fragments. Additionally, environmental datasets derived from metatranscriptomics are often highly complex, fragmented, and biased due to uneven sequencing depth and RNA degradation. Another challenge lies in the limited availability of validated training datasets from non-model organisms, which reduces the accuracy of machine learning-based prediction models. Furthermore, distinguishing functional sRNAs from transcriptional noise remains a key obstacle in environmental samples. Integration of multi-omics datasets, improved secondary structure prediction algorithms, and standardized benchmarking datasets are required to overcome these limitations and improve the reliability of environmental sRNA discovery.

## 5. RNA Interactomes

In bacteria, sRNA-mRNA interactions are indispensable for the rapid adaptation to environmental fluctuation by modulating gene expression in response to perturbations [22]. Most of the sRNAs function by interacting with the target mRNAs, and only a few play their vital regulatory role by interacting with the protein, revealing the importance of RNA-RNA interactome in unveiling the sRNA role. For example, a recent study explored the global RNA-RNA interactome in *Klebsiella pneumoniae* and identified sRNAs related to cell division [113].

Thanks to the advancements in biophysical, biochemical, and cellular techniques, which facilitated the study of RNA regulation by understanding RNA-RNA interactions. Particularly, biophysical techniques have provided the foundation for the detailed analyses of RNA-RNA interactions. For example, the fundamental approach used to reveal RNA-RNA interaction is the Electrophoretic Mobility Shift Assay (EMSA) that utilizes gel electrophoresis to separate RNA complexes based on mobility shifts indicative of interaction. It is one of the simplest methods for studying RNA-RNA interaction. However, it cannot provide the seed region involved in interaction. Similarly, in Surface Plasmon Resonance (SPR) RNA molecule is immobilized on a chip, and its interaction with target RNA molecules is assessed without labeling it [114]. The limitations of SPR include its sensitivity to hydrolysis, and it also cannot reveal detailed interaction localization [115]. Another alternate approach to study RNA interactomes is Single Molecule Forster Resonance Energy Transfer (FRET) [116], which uses a labeled fluorescent dye and offers real-time interaction with target molecules. Co-sedimentation is another approach that involves sucrose gradient fractioning followed by Northern blot analysis to detect RNA pairs that co-sediment, suggesting interaction. Despite its simplicity, its limitation lies in dissociating low-affinity RNA duplexes during the process [117].

Recent advancements in high-throughput sequencing and cross-linking technologies have substantially improved our understanding of the RNA interactome. Techniques, including Cross-linking, Ligation, and Sequencing of Hybrids (CLASH), offer a detailed mapping of RNA interactomes [118]. Similarly, RIA-seq and RAP provide targeted RNA capture followed by sequencing to systematically explore interactions involving specific RNAs, therefore revealing specific regions of interaction [119,120]. Transcriptomics-wide techniques such as SPLASH, LIGR-seq, and Psoralen Analysis of RNA Interactions and Structures (PARIS) [121] have enabled the discovery of numerous novel RRIs, unlocking the complex networks of RNA interactions with high specificity and sensitivity. Likewise, SPLASH uses biotinylated psoralen crosslinking combined with sequencing to dissect complex RNA networks, providing insights into RNA duplex formations [122]. On the contrary, LIGR-seq uses AMT crosslinking followed by circularization and sequencing to enrich and identify RNA duplexes, offering rapid identification of RNA interactions [123]. MARIO extends these capabilities to include RNA-protein interactions, offering a comprehensive view of the interactome mediated by both RNA and associated proteins [124]. These methods facilitate revealing complex networks of RNA-based regulatory mechanisms, highlighting their key roles in gene expression and cellular functionality [125].

The transition from experimental to computational techniques in studying RNA-RNA interactions reveals an equally complex field where multiple computational tools have been developed over the years for accurately predicting these interactions. Recent studies discussed four main strategies employed by various tools including interaction-based, accessibility-based, concatenation-based, and complex joint structure prediction methods [126], for studying RNA interactomes. Integrating the computational methods with experimental data from techniques like CLASH and PARIS can significantly enhance the accuracy and reliability of RNA-RNA interaction predictions.

### 5.1. Interaction-Based Prediction Tools

GUUGle [127], RNAplex [128], RIsearch [129], and RNAduplex [130] are the widely used tools for predicting RNA-RNA interaction, which work by predicting intermolecular base pairs without considering intramolecular interactions. GUUGle identifies ungapped interactions exceeding a user-defined length, utilizing Gibbs free energy calculations for scoring, thus serving as a fundamental baseline in performance evaluation. RNAplex and RIsearch extend this paradigm by estimating the minimal free energy of binding, albeit without incorporating the influence of intramolecular structures, thereby prioritizing computational efficiency over comprehensive structural consideration.

### 5.2. Accessibility-Based Prediction Tools

IntaRNA is a leading tool in this category, which incorporates the accessibility of RNA regions to interactions [131]. It considers both the potential for intermolecular pairing and the likelihood that these sites are accessible and not involved in intramolecular base pairing. This makes IntaRNA highly suitable for predicting interactions in scenarios where RNA molecules may have a significant internal structure that affects their interaction potential. RNAup [132] functions by assessing RNA secondary structure on the accessibility of interaction sites, thereby offering a better understanding of interaction dynamics influenced by RNA conformation and providing a balance between interaction energy and accessibility.

### 5.3. Concatenation-Based Prediction Tools

Pairfold [133] and RNAcofold [130] assess RNA-RNA interaction prediction by concatenating the sequences of two RNAs and applying standard secondary structure prediction algorithms. This method allows them to predict joint structures that may not accurately reflect complex RNA structures that involve long-range interactions or pseudoknots. These tools are considered more effective for predicting simple interactions but are not useful for revealing structurally complex interactions.

### 5.4. Complex Joint Structure Prediction Tools

RactIP uses integer programming to address both intra- and inter-molecular interactions comprehensively, making it suitable for cases where the interaction involves complex structures or where multiple competing interactions might occur [134]. It is particularly effective for longer RNA sequences where the complexity of potential interactions increases. PETcofold involves integrating evolutionary conservation data from multiple sequence alignments, thus enhancing the predictive accuracy for conserved interactions among diverse RNA species [135].

### 5.5. Role of Hfq and ProQ in RNA-RNA Interactions

The activity and stability of bacterial sRNAs are critically dependent on their interactions with a suite of RNA-binding proteins (RBPs). As comprehensively reviewed by Quendera et al. [68], these RBPs include not only the canonical RNA chaperones Hfq and ProQ, which facilitate sRNA-mRNA base pairing, but also the global regulator CsrA and various ribonucleases (RNases) that govern sRNA turnover and processing. The interplay between these proteins determines the fate of sRNAs from their biogenesis to degradation, forming an intricate network that ensures precise post-transcriptional regulation. Hfq and ProQ are pivotal in facilitating and regulating RNA-RNA interactions within bacterial cells, primarily acting as chaperones that stabilize sRNAs and promote the binding with specific mRNA targets. This interaction is crucial for controlling the stability of mRNAs and their translation, thereby influencing cellular response mechanisms. Hfq has been extensively studied for its role in stabilizing sRNAs and in promoting the interaction with target mRNAs [136]. Recent findings in *Pseudomonas aeruginosa* reveal that Hfq mediates an intricate network of sRNA-mRNA interactions, significantly expanding the known regulatory scope of sRNAs in this pathogen. This study conducted using RIL-seq (RNA interaction through ligation and sequencing) highlighted that Hfq-associated sRNAs, including PhrS, can interact with a surprisingly large number of mRNA targets, thereby influencing key pathways such as quorum sensing and virulence factor production [137]. This interaction pattern underscores the adaptability of sRNAs in regulating diverse cellular functions through a mechanism that might be common across various bacterial species. While Hfq is a central RNA chaperone in Gram-negative bacteria, its role in Gram-positive species is more limited. As reviewed by Brantl and Haq [10], in *Bacillus subtilis*, Hfq binds only ~150 transcripts and does not play a decisive role in sRNA-mRNA interactions, instead acting as a fine-tuning regulator in the stationary phase. By contrast, the small RNA chaperone CsrA (74 aa) promotes the interaction between the sRNA SR1 and its target *ahrC* mRNA in *B. subtilis*, demonstrating that different Gram-positive bacteria may employ alternative RNA-binding proteins for sRNA-mediated regulation. ProQ has emerged as another pivotal RNA-binding protein, noted for its role in different bacteria, somewhat overlapping functions with Hfq [138]. The identification of ProQ as a major RNA-binding protein was greatly facilitated by Grad-seq (gradient profiling by sequencing), a technology that simultaneously profiles the sedimentation of RNAs and proteins through glycerol gradients to predict stable RNA-protein complexes [139]. This approach, applied to *E. coli* by Hör et al. [139], not only confirmed the complex formation of known sRNA-binding proteins (CsrA, Hfq, ProQ) with their RNA partners but also revealed unexpected ribosome associations of several sRNAs and enabled the discovery of new ribosome-associated proteins like YggL, demonstrating the power of this technology for uncovering RNA-protein interactions on a global scale. ProQ binding affinities and specificities have been elucidated through RNA immunoprecipitation coupled with high-throughput sequencing (RIP-seq) [140]. ProQ binds with a higher affinity to RNA molecules and shows a preference for different subsets of RNAs, including those involved in osmotic and cell envelope stress responses. The interaction dynamics between ProQ and Hfq reveal a complex interplay where both proteins can share and compete for RNA targets. This competition and redundancy add layers of regulation, suggesting a mechanism of RNA-mediated gene regulation that ensures cellular resilience and adaptability. Integrating these insights with advanced experimental methodologies such as CLASH and PARIS allows for a more refined exploration of the RNA interactome. These methods, complemented by bioinformatics tools, facilitate a comprehensive mapping of the interactions and the regulatory impacts of Hfq and ProQ, enabling a deeper understanding of the molecular mechanism of bacterial gene regulation. Recent advances in Grad-seq and RIP-seq have significantly expanded the concept of the RNA interactome beyond classical sRNA–mRNA pairing. Grad-seq enables the global fractionation of RNA–protein complexes based on their sedimentation profiles, allowing the identification of previously unknown RNA-associated protein assemblies and higher-order ribonucleoprotein complexes. In contrast, RIP-seq provides transcriptome-wide mapping of RNAs directly associated with specific RNA-binding proteins such as Hfq and ProQ under native cellular conditions. Together, these approaches have revealed that bacterial RNA regulatory networks are far more interconnected than previously assumed, involving extensive RNA–protein, RNA–ribosome, and multi-RNA complex interactions. This has shifted the understanding of the RNA interactome from simple one-to-one regulatory pairs toward a dynamic and multilayered regulatory network that integrates post-transcriptional regulation at a systems level. Despite their power, RNA interactome technologies such as CLASH, PARIS, SPLASH, RIL-seq, Grad-seq, and RIP-seq have inherent limitations. Crosslinking-based methods may bias toward stable, abundant interactions, while transient or low-affinity interactions can be missed. Scalability is limited in complex microbial communities, and experimental noise or ligation artifacts can generate false positives. Furthermore, applying these techniques to environmental microbes is challenging due to low biomass, community complexity, and difficulty capturing dynamic interactions in natural conditions. Awareness of these limitations is essential for interpreting RNA interactome data and designing robust experiments.

## 6. Applications of sRNAs

Recent evidence demonstrates that sRNAs regulate nearly all cellular responses in prokaryotes [141]. Consequently, the design of novel sRNAs as tools for metabolic engineering has gained considerable attention. Because of their advantages over other regulatory systems, including cost-effectiveness, small size [142] and rapid response to environmental signals, sRNAs have been utilized as essential tools in metabolic engineering and synthetic biology. The researchers have used artificially synthesized sRNAs to mimic bacterial gene regulation. For example, in a genetic circuit engineering study by Coleman et al. [143], sRNAs were designed to negatively regulate the expression of specific genes. In this study, the authors modified the naturally occurring MicF sRNA by artificially inserting the complementary sequences of ribosome regions of their target genes between two stem-loop structures of sRNA. Advances in emerging technologies related to RNA biology have facilitated the design of artificial sRNAs for specific gene regulation [144]. For example, Man et al. have devised a strategy for designing artificial sRNAs to inhibit the expression of a specific gene in bacteria [145]. Rodrigo et al. designed an automated approach for sRNA artificial design and characterization in cellular environments [146]. Another study designed and utilized the synthetic sRNAs to increase the yield of the S-adenosylmethionine by modulating the concentration of ATP in *E. coli* [147]. Miao et al. designed an artificial sRNA named anti4, which suppresses the expression of *glnR* and leads to the enhanced production of nisin in *Lactococcus lactis* F44 [148]. Similarly, synthetic sRNAs have been designed and successfully utilized for regulating the carbon flux in cyanobacteria to use them as green microbial factories [149]. Recently, a known sRNA SR6, was modified, and the synthetic sRNA showed Hfq-independent and efficient regulation of the target gene compared to native sRNA [150]. sRNAs have therefore emerged as powerful tools for application to metabolic engineering and synthetic biology. The potential of sRNAs as metabolic engineering targets extends to industrial antibiotic production. Caicedo-Montoya et al. [151] identified two sRNAs in *Streptomyces clavuligerus* that are predicted to interact with genes in the clavulanic acid (CA) biosynthetic cluster. One sRNA (“p_RNA_R_6021554-6021232-”) was downregulated during peak CA production and is proposed to negatively regulate the pathway, possibly through interaction with the global regulator *relA*. The authors suggest that deleting or inactivating such sRNAs could depress target genes and enhance CA yields, representing a novel approach to strain improvement beyond traditional transcriptional regulator engineering.

The sRNAs as a tool in synthetic biology and metabolic engineering have gained much attention during the last few years, and extensive work has been done. For example, anhydrotetracycline (aTc)-inducible synthetic sRNA system previously found in *Rhodopseudomonas palustris* was used in *E. coli* strain to downregulate the expression of *sucC*, *aceA*, and *hemB* and shift the metabolic flux of succinyl-CoA towards 5-Aminolevulinic acid (5-ALA) biosynthesis [152]. 5-ALA has wide applications commercially in contemporary farming and medicine, being a promising biostimulant, feed ingredient, and photodynamic drug [153,154]. Tanniche et al. designed non-Hfq-dependent modified sRNAs that bind to the selective mRNAs and improve their stability, ultimately leading to upregulation of their gene expression [155]. In another study, Liu et al. designed two artificial RNA scaffold structures (0D and 2D scaffolds) to co-localize enzymes of the mevalonate pathway in *E. coli*. This approach resulted in significant quantitative improvements, i.e., mevalonate production increased by 84.1% (reaching 3.13 ± 0.03 g/L) with the 0D scaffold and by 76.5% (3.00 ± 0.09 g/L) with the 2D scaffold compared to the control strain lacking RNA scaffolds. Furthermore, when the 0D scaffold was applied to co-localize the enzymes MvaE and MvaS for isoprene production, yields reached 609.3 ± 57.9 mg/L, a 142% increase over the non-scaffold strain [156]. The sRNA PrrF1 scaffold encoded by the *Pseudomonas aeruginosa* was engineered in *Halomonas bluephagenesis*, which, by binding to its target gene *prpC*, demonstrated about 7-fold enhanced content of 3-hydroxyvalerate in poly(3-hydroxybutyrate-co-3-hydroxyvalerate) [157]. Another study reported the design and application of sRNAs that target riboswitches and upregulate the gene expression (rtRNA) and engineered *E. coli* chassis for the enhanced production of riboflavin [158]. The well-known sRNA RyhB was overexpressed in *Enterobacter aerogenes* which by downregulating the formate dehydrogenase led to shift the metabolic flux towards 2,3-Butanediol production which is a potential biofuel [159]. Yang et al. engineered *E. coli* strains harboring multiple synthetic sRNAs capable of downregulating the target genes at genome-scale and developed strains capable of enhanced production of compounds, including L-proline, L-threonine, crude violacein, and indigo [160].

A study by Na and coworkers reported anti-*tyrR* and anti-*csrA* sRNAs, which can enhance tyrosine production in *Escherichia coli*. The combinatorial knockdown of the *tyrR* and *csrA* genes, which encode transcriptional regulators that typically repress tyrosine biosynthesis, redirects the metabolic flux, leading to a significant increase in tyrosine production [161]. Additionally, the anti-*murE* sRNA targets the *murE* gene, increasing cadaverine production by 55%, an important precursor for nylon synthesis [162]. In a study by Kang and coworkers, synthetic sRNA RyhB was utilized to downregulate *sdhCDAB*, resulting in a significant accumulation of succinic acid and acetic acid [163]. Furthermore, the overexpression of SgrS sRNA in *E.coli* K-12 has demonstrated a significant reduction in acetate secretion by targeting the *ptsG* gene [164]. Recently, a study utilized a synthetic sRNAs targeting the *acrA* gene in *Escherichia coli* leading to increased sensitivity to β-lactam antibiotics, showcasing the potential of these sRNAs in enhancing antibiotic efficacy [165]. The previous work related to the application of sRNAs in synthetic biology and metabolic studies during the recent past has been discussed in Table 4.

**Figure 3:**
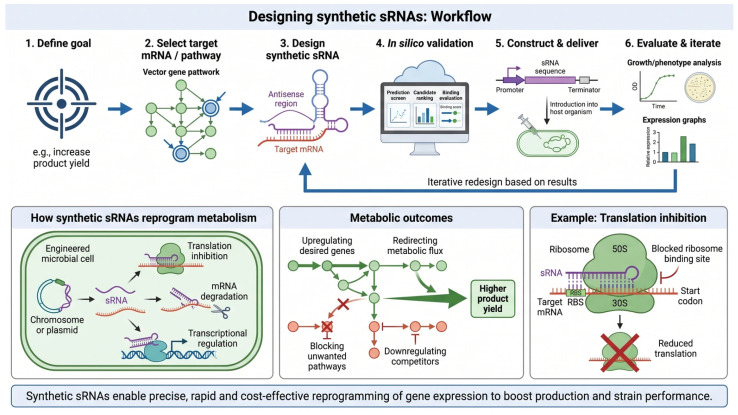
Using customized synthetic sRNAs for application in metabolic engineering.

The development of sRNA databases should help broaden the applicability of the increasing number of identified sRNAs [168]. Moreover, various algorithms have been proposed for designing sRNAs with desired structures [169,170]. It is suggested to design synthetic sRNAs for modular and global regulation of selective gene expression in future studies. Developing engineered bacterial strains using sRNAs to shift the metabolic flux is advantageous over conventional metabolic engineering strategies due to small size, easy manipulations, rapid response, and cost-effectiveness (Figure 3). Certain sRNAs and associated proteins undergo phase separation to form membraneless condensates, which spatially organize regulatory networks within the cell. In addition, CRISPR RNAs have roles beyond immune defense, contributing to the regulation of gene expression and metabolic pathways in prokaryotes, revealing multifunctional regulatory strategies. In marine and environmental biotechnology, sRNA-based regulatory systems offer promising strategies for engineering stress-resistant microbial strains capable of surviving extreme environmental conditions such as salinity, temperature variation, and nutrient limitation.

In summary, the integration of computational predictions with high-throughput experimental methods has significantly expanded the repertoire of known sRNAs, revealing their widespread occurrence and diverse mechanisms of action across bacterial lineages. Notably, the recent metagenomic and metatranscriptomic studies have highlighted the vast and previously underappreciated functional diversity of environmental sRNAs in uncultured microbial communities, underscoring their critical ecological significance and roles in microbial adaptation to diverse environments. These advances have not only advanced our understanding of microbial gene regulation but also opened new horizons for applications in metabolic engineering and synthetic biology. However, key challenges remain, including the need for improved computational tools, more comprehensive functional characterization, and an expanded exploration of the regulatory networks and mechanisms governing sRNAs, particularly in underexplored microbes, including uncultured ones. Furthermore, the potential of sRNAs to mediate host–pathogen interactions offers promising avenues for novel therapeutic strategies. As research continues to unveil the intricate roles of sRNAs in prokaryotic biology, their application in biotechnology and environmental science is expected to make significant contributions, shaping the future of gene regulation research. In comparison with other synthetic gene regulation tools such as CRISPR interference (CRISPRi) and riboswitch-based systems, sRNA-mediated regulation offers distinct advantages in terms of tunability, reversibility, and reduced metabolic burden. Unlike CRISPRi, which relies on stable dCas9–DNA binding and may exhibit persistent repression, sRNA-based knockdown operates at the post-transcriptional level and allows rapid and reversible regulation of gene expression. Additionally, sRNAs impose minimal cellular burden due to their small size and do not require heterologous protein expression, in contrast to CRISPRi systems that depend on continuous dCas9 production. Compared to riboswitches, sRNAs provide greater flexibility in target design and can be engineered to regulate multiple genes simultaneously with fine-tuned expression control. However, sRNA-based regulation may exhibit variability in knockdown efficiency depending on RNA stability and target accessibility, whereas CRISPRi generally provides stronger and more stable repression. Overall, sRNAs represent a highly flexible and low-burden regulatory tool that is particularly suitable for dynamic metabolic engineering applications where reversible and adjustable gene control is required.

## Figures and Tables

**Table 1:** Versatile functions of sRNAs in diverse bacterial species.

sRNA Function	sRNA Name	Target Genes	Required Factors	Reference
***Cell division***				
*Rhodobacter sphaeroides*	StsR	*dcw* gene cluster	UpsM	[42]
*Chlamydia trachomatis* EB	IhtA	*hctA* and *ddbA*		[43]
***Photosynthesis***				
*Rhodobacter sphaeroides*	PcrX	*pufLMX*	Hfq	[44]
*Synechocystis 6803*	IsaR1	upp, *crtH*		[45]
***Amino acid metabolism***				
*Pseudomonas aeruginosa*	PrrF	*ldh*, *bkdA1*, *bkdB*, *lLiuD*, and *liuE*		[46]
*Listeria monocytogenes*	Rli47	*ilvA*	σ^B^	[47]
***Carbon metabolism***				
*Escherichia coli*	Spot42	*galK*	Hfq	[48]
	CsrB–CsrC	csrA	CsrD, RNase E,	[49]
*Salmonella enterica*	DsrA	*pflB*	RNase E	[50]
*Pseudomonas aeruginosa*	RsmY–RsmZ	*rsmA*	PNPase	[51]
***Iron & potassium regulation***				
*P. aeruginosa*	RsmY and RsmZ	*T6SS*	PqsA, hsiA2	[52]
*Mycobacterium tuberculosis*	Mcr11	*oprA*		[53]
***Quorum sensing and biofilm formation***				
*P. aeruginosa*	RhlS	*rhlI*, *fpvA*		[54]
	PhrD	*rhlR*		[55]
	AmiL	*las*/*rhl*, *rhlI*		[56]
*E. coli*	CsrB/C	*csrA*		[57]
	McaS	*flhDC*		
	MicA	*ompA*	σ^E^	[58]
***Cold adaptation***				
*Shewanella piezotolerans* WP3	cis4, cis5, cis6, cis10, and cis15	*ompA*, *Amidohydrolase*, *dioxygenase*, PAS		[7]
***Acid stress response***				
*E. coli*	RyjB	*phoP*		[59]
	6S RNA	*pspF*		[60]
***Oxidative stress response***				
*Staphylococcus aureus*	RsaC	*soda*, *mnt*ABC	RNase III	[61]
***Osmotic stress***				
*E. coli*	ryfA	*rpoH*	σ^32^	[62]
	RprA	*rpoS*	Hfq, RNase III	[63]
***Aerobic*** **/** ***anaerobic conditions***				
*Sphingopyxis granuli*	FnrS	*fnr*		[64]
*E. coli*	OmrA/OmrB	Four porins	Hfq	[65]
*E. coli*	RybB	*omp*	σ^E^	[66]
*E. coli*	RprA	*rpoS*	Hfq	[63]
***Virulence***				
*Salmonella typhimurium*	STnc150	*fimA*		[67]
*Mycobacterium tuberculosis*	MTS1338	*furA*, *whiB4*, *phoP*		[68]
*Streptococcus suis*	sRNA23	FBA/*rplB*		[69]
*Shigella dysenteriae*	RyfA1	*ompC*	Hfq	[70]
*Streptococcus pyogenes*	FasX	*fasBCA*		[71]
*Clostridium perfringens*	RivX	*mga*	*rivR*	[72]
*P. aeruginosa*	RsmY–RsmZ	*T3SS*, *pel*	HptB/RetS	[73]
***Specific stress phenotypes linked to 6S RNA deletion***				
*Rhodobacter sphaeroides*	6S RNA	*sspA*	--	[39]
*Escherichia coli*	6S RNA	*soxS*, *ahpC*, *sodA*, *tpx*	--	[40]
*Bacillus subtilis*	6S-1 RNA, 6S-2 RNA	Multiple stress-related proteins	--	[41]

**Table 2:** Summary of the environmental sRNA in varied habitats.

Sr. #	Number of sRNAs	Source of Isolation	Techniques	Description	Reference
1	Hundreds (228 groups)	Deep-sea hydrothermal sediment, Guaymas Basin	Metagenome and metatranscriptome	Regulate microbial activity in deep-sea environments	[79]
2	24 sRNAs	Oceanic environmental populations	Metatranscriptomic	sRNA Yfr28 stimulated under low-nitrogen conditions	[80]
3	2181sRNAs	Oceanfront Deep-Subsurface Hot Spring	Metatranscriptomic	tRNA anticodon relationships and degradation sites	[81]
4	18,000 to 47,000 sRNAs (66 groups)	HOT Station ALOHA at various depths	Metatranscriptomics	Depth-dependent psRNA variations, carbon metabolism	[82]
5	12,097 sRNAs	Human oral samples	Metatranscriptomics	Regulate oral microbiome transition from commensal to dysbiotic	[83]
6	19 sRNAs	Blood plasma	Illumina and sRNA isolation columns	Non-human sRNA species in column eluates	[84]
7	208 candidate sRNAs	Human gut	Metagenomic and metatranscriptomic	Differences in gene expression in vitro vs. in vivo	[85]
8	59 sRNAs	Human gut	Metatranscriptomic	Insights into gut microbiota functionality and regulation	[86]
9	155 conserved, 925 antisense, 613 intergenic sRNAs	Halite nodules, Atacama Desert	Metatranscriptomics	Functional roles and mechanisms of sRNAs in extremophiles	[87]

**Table 3:** Summary of results of bioinformatics identification and verification of bacterial sRNAs.

Targeted Species of Bacteria	Bioinformatics Methods Used	Validation Method	Total sRNAs Identified			Validation Rate (%)	Reference
			Detected	Tested	Validated		
*Pseudomonas aeruginosa*	sRNAPredict2	Northern blotting	2759	31	17	54.8	[102]
*Staphylococcus aureus*	NAPP	Northern blotting	189	24	7	29.2	[103]
*Synechocystis* PCC6803	RNAz	Northern blotting	383	2	2	100	[104]
*Burkholderia pseudomallei*	SIPHT, sRNAScanner & RNA Infernal	RT-PCR	1306	15	8	53.3	[92]
*Burkholderia cenocepacia* J2315	RNAalifold & TransTermHP	Northern blotting	NA	24	24	100	[105]
*Brucella abortus* 2308	SIPHT & NAPP	RT-PCR	129	20	7	35	[106]
*Shewanella piezotolerans* WP3	RNAz & RNA Infernal	RT-qPCR	209	16	15	93.8	[7]
*Proteus mirabilis*	Comparative analysis	Northern blotting	14	6	6	100	[107]
*Streptococcus pneumoniae* D3	RNA seq analysis	Northern blotting	66	66	30	45.5	[108]
*Bacteroides thetaiotaomicron*	ANNOgesic	Northern blotting	49	11	9	81.8	[109]
*Prochlorococcus* NATL2A	RNAz	Northern blotting	389	3	3	100	[80]
*Prochlorococcus* MED4	RNAz	Northern blotting	982	2	2	100	[80]
*Bordetella pertussis*	ANNOgesic	Northern blotting	143	10	10	100	[110]
*Streptococcus mutans*	RNAalifold, ARNold, and TransTermHP	Northern blotting	15	15	15	100	[111]
*Histophilus somni* 2336	BPROM, ARNold, RNAfold	Northern blotting	8	3	3	100	[112]

**Table 4:** sRNAs targeted as tool having application in synthetic biology and metabolic engineering in recent past.

Sr. #	sRNA Name (If Given)	Regulatory Gene	Targeted Bacteria	Enhanced Product	Quantitative Increase	Reference
1	Anhydrotetracycline (aTc)-inducible synthetic sRNA	sucC, aceA, and hemB	*Escherichia coli*	5-Aminolevulinic acid	↑26.8% titer (1.56 g/L), ↑38.1% yield	[152]
2	asB606	amCyan	*Escherichia coli*	Diaphorase	↑50–80% expression	[155]
3	0D RNA scaffolds and 2D RNA scaffolds	mvaE and mvaS	*Escherichia coli*	Mevalonate and Isoprene	↑84.1%/76.5% mevalonate; ↑142% isoprene	[156]
4	PrrF1	prpC	*Halomonas bluephagenesis*	3-hydroxyvalerate	3.1→21 mol% (~6.8-fold)	[157]
5	Synthetic rtRNAs	purE, xpt, nupG, pbuE, and ribDG	*Escherichia coli*	Riboflavin	↑up to 103-fold expression	[158]
6	RyhB	Formate dehydrogenase	*Enterobacter aerogens*	2,3-Butanediol	↑~7-fold titer	[159]
7	MicC	acnB and sdhB	*Pseudomonas putida*	Modulate the gene expression	↓40%/20% enzyme activity	[166]
8	MicC	pyk, ldhA, and odhA	*Corynebacterium glutamicum*	Glutamate	↑~3-fold titer & yield	[167]
9	Multiple synthetic sRNAs	61 E. coli genes	*Escherichia coli*	L-proline	33.8 ± 1.6 g/L	[160]
10	Anti-tyrR, Anti-csrA	tyrR, csrA	*Escherichia coli* S17-1	Tyrosine	2 g/L titer achieved	[161]
11	Anti-murE	murE	*Escherichia coli*	Cadaverine	↑55% vs. reported strain	[162]

## Data Availability

No new data were created or analyzed in this study. Data sharing is not applicable to this article.
